# Interpersonal Trauma and Risk of Depression Among Adolescents: The Mediating and Moderating Effect of Interpersonal Relationship and Physical Exercise

**DOI:** 10.3389/fpsyt.2020.00194

**Published:** 2020-04-15

**Authors:** Runsen Chen, Ke Peng, Jianbo Liu, Amanda Wilson, Yuanyuan Wang, Meredith R. Wilkinon, Siying Wen, Xiaolan Cao, Jianping Lu

**Affiliations:** ^1^Department of Child Psychiatry of Shenzhen Kangning Hospital, Shenzhen Mental Health Center, Shenzhen University, Mental Health School, Shenzhen, China; ^2^The George Institute for Global Health, UNSW, Sydney, NSW, Australia; ^3^School of Public Health, The University of Sydney, Sydney, NSW, Australia; ^4^Division of Psychology, Faculty of Health and Life Sciences, De Montfort University, Leicester, United Kingdom

**Keywords:** trauma, exercise, interpersonal, depression, adolescent

## Abstract

Interpersonal trauma in adolescent is an important public health concern. Depression can be a main consequence of interpersonal trauma, which subsequently results in various negative mental health outcomes. Previous research has investigated the independent effects of interpersonal trauma, interpersonal relationships and physical exercise on the risk of depression. However, the interaction effect of the three factors on depression remains unclear. We aim to investigate the associations between these potential factors and depression in adolescents, and explore the interaction effect of the three aforementioned factors. A cross-sectional study was conducted in Shenzhen, China, in 2017. A total of 1,883 adolescents from 11 middle schools and high schools were recruited. Demographic information, depressive symptoms, physical exercise, interpersonal relationships, interpersonal trauma, and academic record were collected through the use of standardized questionnaires. A linear regression model was performed to explore the association between these variables and depression. Pathway analysis was used to explore the role of potential mediators and moderators. The results showed that interpersonal trauma and poorer interpersonal relationships were significantly associated with depression (*p* < 0.05). We identified a mediating role of interpersonal relationships in the relationship between interpersonal trauma and depression, and a moderating role of physical exercise between interpersonal trauma and interpersonal relationships. This is the first study to examine the interaction effects of interpersonal trauma, interpersonal relationships and physical exercise on depression in adolescents. The current study therefore provides insights into factors which impact the mental health of adolescents. Through examining these factors one can gain further insight into potential factors associated with depression and therefore then develop more tailored interventions in order to support adolescents' mental well-being.

## Introduction

Exposure to interpersonal trauma is common and has become an important public health concern in adolescence. It can be defined as an event experienced by the individual such as physical, emotional, or sexual abuse (1, 2). Previous research has reported that two thirds of adolescents have had at least once experience of interpersonal trauma in some form (3). Depression is considered one of the main consequences of interpersonal trauma and can result in further negative mental health outcomes for the individual. A number of studies have reported significant relationships between interpersonal trauma and mental health outcome (4–7), and the treatment of interpersonal trauma is found to be more costly and likely to cause mental disorders in comparison to non-interpersonal trauma for adolescents (3, 8). In the United States (US), the cost of hospitalization for child-abuse was found to be 9 times higher than for those adolescents who reported not experiencing child-abuse (9). It is estimated that the cost for one incident of childhood abuse is $4,397 in the US.

Rapid physical and biological changes, as well as rapid psychological development are observed during adolescence. This is especially pertinent in the present day when social relationships have a significant role on adolescents' mental health. Social relationships with family members, peers and teachers play an important role in building resilience of adolescents (10, 11), and having a stable and supportive family environment provides emotional and physical safety, as well as well-being to adolescents (12). Furthermore, having a positive relationship with family members has been deemed an indicator of a healthy family. This is supported by research that has found an inversed association between risk of depression and good family relationships (12), suggesting that having a healthy relationship with one's family can serve as a protective factor for depression. Direct effects of a dysfunctional family environment on incidents of depression in adolescents have also been documented (12–15). Relationships with peers and teachers at school are another critical factor during the psychological development of children. Similarly, high levels of support from teachers was found to reduce the risk of depression of students and have a positive effect on their academic achievements (16–18). Moreover, peer victimization was found to be a predictor of depression later on in adolescence (19, 20). These findings are unsurprising since adolescents' social network can influence one's mental health (21).

Regular physical exercise has been proposed as an effective intervention for mental disorders. Previous research has indicated a positive impact of physical exercise on depression amongst children and adolescents. A systematic review and meta-analysis conducted by Larun et al. (22) found that exercise as an intervention had a significant but small effect on reducing depression and anxiety scores in children and adolescents. Similarly, a small but significant overall protective effect of physical exercise on depression in adolescents were found in another systematic review and meta-analysis study conducted by Brown and colleagues (23). These findings suggest there may be an impact of physical exercise on adolescents' mental health.

In China, the prevalence of depressive symptoms in children and adolescents was reported at 19.85% in a meta-analysis of 18 studies with a sample size of 29,626 participants (24). This prevalence of depression in Chinese adolescents is reportedly higher when compared on a global level (25). Another study reported that 1.3% of Chinese children and adolescents have major depressive disorder, which further supports higher rates of depression when compared to the worldwide figures (26). Risk factors of depression among Chinese adolescents have been documented and can be divided into four categories; family factors, social factors, socio-demographic factors, and physical factors (27). However, compared to Western countries, very few prevention interventions have been implemented to manage depression among children and adolescents in China (28). A school-based health promotion program in China reportedly reduced depressive symptoms at the post-intervention phase, which indicated that a healthier school climate, as well as a healthier social and emotional environment could reduce the risk of depression (29). Therefore, further intervention studies need to be developed to continue to test whether school based interventions can improve the circumstance of mental health in children and adolescents in China. This is especially important given that such interventions have been developed successfully in both China (29) and other countries such as the United Kingdom (UK) (30).

In order to develop prevention and intervention for depression, it is important to understand the factors associated with depression in adolescents in China. Although the independent effects of interpersonal trauma, interpersonal relationships and physical exercise as a risk of depression have been investigated previously, the association between physical exercise and interpersonal relationships and the interaction effect of these three factors on depression remains unclear. The purpose of this research is to examine these factors within a single study for the first time. Based on the previous results and inconsideration of the protective effect of positive interpersonal relationships and physical exercise on depression, we hypothesized that the association between interpersonal trauma and depressive symptoms would be attenuated among adolescents with positive interpersonal relationships and who participated in regular physical exercise.

## Method

### Participants

The present study was a cross-sectional study using a three-stage convenience sampling design conducted in Shenzhen City, China in 2017 (31). Two central districts (Luohu & Futian) were selected to conduct the study in stage one. A total of 11 middle schools (grade 7–9) and high schools (grade 10–12) in the two study districts were then randomly selected in stage two in an attempt to reduce researcher bias. In stage three, eight classes of each grade were randomly selected to again reduce bias and provide a large enough sample for the analysis.

### Data Collection

All participants completed a self-reported questionnaire anonymously. The participants were advised their answers would be confidential and that their specific answers would not be shared with their respective school or teachers. The participants answered the questionnaire with the advice of trained school counselors upon request to ensure the accuracy of their answers. Ethics approval was obtained from the Ethics Committees of Shenzhen Mental Health Center.

### Measure

#### Demographic Variables

The following demographic information was collected: sex, age, and academic record, and whether the participant was the only-child in the family.

#### Depression

Depression was assessed by the Chinese version of the Center for Epidemiologic Studies-Depression Scale (CES-D) which has been validated among Chinese adolescents (32). The depression score was calculated by summing the scores (ranging from 0–60), with a higher score indicating more severe depression.

#### Physical Activity

Participants who self-reported regularly participating in physical activities were classified as part of the physical exercise engaged group. Those that did not self-report any form of exercise were then classified as the non-engaged group.

#### Interpersonal Relationship

Interpersonal relationships were measured by four questions: 1) “How do you perceive your interpersonal relationships with your peers?” Response categories ranged from 1 (good) to 3 (bad); 2) “How do you perceive your interpersonal relationships with your teachers?” Response categories ranged from 1 (good) to 3 (bad); 3) “How do you perceive your interpersonal relationships with your family members?” Response categories ranged from 1 (good) to 3 (bad) and 4) “Are you happy/ satisfied with your interpersonal relationships?” Response categories ranged from 1 (very satisfied) to 5 (very dissatisfied). The questions in this study have been previously used to assess the interpersonal relationships among Chinese adolescents in other studies (33, 34). Interpersonal relationships were revealed by the total score (ranging from 4 to 12), with a higher score indicating the adolescent had poor interpersonal relationships.

#### Interpersonal Trauma

Interpersonal trauma was measured by the following three questions: 1) “Have you had any physical abuse by your family, or others, in the past 6 months? For example, being neglected by others, experiencing violence?”; 2) “Have you had any verbal abuse by your family, or others, in the past 6 months? For example, being called by an offensive nick name, being criticized by others?” Participants who reported yes to any of these questions were classified as having interpersonal trauma.

#### Statistical Analysis

Descriptive statistics were used to summarize the characteristics of participants. Univariate linear regression was performed to assess the association between these potential factors and risk of depression. The potential factors included: age, sex, being the only-child, physical exercise, academic record, and interpersonal relationship. Multivariate linear regressions were performed to investigate the associations between interpersonal trauma, interpersonal relationships, physical exercise and risk of depression. Variables that were significantly associated with depression in the univariate analyses were entered into the final multivariate linear model. Data was analyzed with the Statistical Package for Social Sciences (SPSS) V.22.0. In order to provide a more functional understanding of the interacting relationships among interpersonal trauma, interpersonal relationships, physical exercise, and depression, pathway analysis was used to explore the role of potential mediator (interpersonal relationships) and moderator (physical exercise) using PROCESS (35). A bootstrapping method (a bootstrap sample with 5,000 was performed) was used to examine the indirect mediating and moderating effects. Bootstrapping was done at the 95% confidence interval and statistical significance was calculated at the *p*-value 0.05 in order to be considered indirect meditating and moderating effects (36).

## Results

A total sample of 2,200 potential participants completed questionnaires, the response rate was of 95.6%. We excluded participants who had missing, inconsistent or implausible value in the responses leaving 1,883 of participants for the final analyses. Among the 1,883 adolescents, 945 (50.2%) were males and 910 (48.3%) were females; the mean (SD) age was 15.26 (1.63) years; and 36.8% were the only child in the family. Most participants (90.4%) reported to engage in the physical exercise and 51.6% of all had experience of interpersonal trauma. The median score of interpersonal relationship was 7.0 (interquartile range 5–8) and the median score of depression was 13.0 (interquartile range 7–21).

The results of the univariate analyses are shown in Table 1. Age, sex, physical exercise and academic record were significantly associated with the risk of depression. The results of the multivariate regression models are shown in Table 2. In models 1 and 2, analysis was performed to examine the relationship between interpersonal trauma, interpersonal relationships and depression without adjustment to demographic variables. Interpersonal trauma and poorer interpersonal relationships were significantly associated with the risk of depression. In model 3, after adjustment for age, sex and academic record, a lack of physical exercise was significantly associated with depression.

**Table 1:** Factor associated with risk of depression in univariate regression models.

	**Unstandardized coefficients**		**Standardized coefficients**	**t**	**Sig**.
	**B**	**Std. Error**	**Beta**		
Age	0.484	0.144	0.078	3.362	0.001
Sex	−1.203	0.474	−0.059	−2.537	0.011
Only child	0.150	0.498	0.007	0.302	0.763
Physical exercise	−6.623	0.786	−0.191	−8.421	<0.001
Academic record	1.812	0.192	0.215	9.461	<0.001
Interpesonal relationships	2.894	0.105	0.536	27.545	<0.001

**Table 2:** The results of multivariate regression models.

Model		**Unstandardized coefficients**		**Standardized coefficients**	**t**	**Sig**.
		**B**	**Std. Error**	**Beta**		
Model 1	Interpersonal trauma	4.186	0.461	0.205	9.073	<0.001
Model 2	Interpersonal trauma	2.556	0.398	0.125	6.415	<0.001
	Interpersonal relationships	2.790	0.105	0.517	26.514	<0.001
Model 3	Interpersonal trauma	2.916	0.410	0.145	7.119	<0.001
	Interpersonal relationships	2.499	0.111	0.465	22.528	<0.001
	Age	0.239	0.126	0.039	1.901	0.057
	Sex	−0.994	0.396	−0.049	−2.510	0.012
	Physical exercise	−3.240	0.688	−0.095	−4.711	<0.001
	Academic record	0.827	0.167	0.099	4.957	<0.001

**Figure 1:**
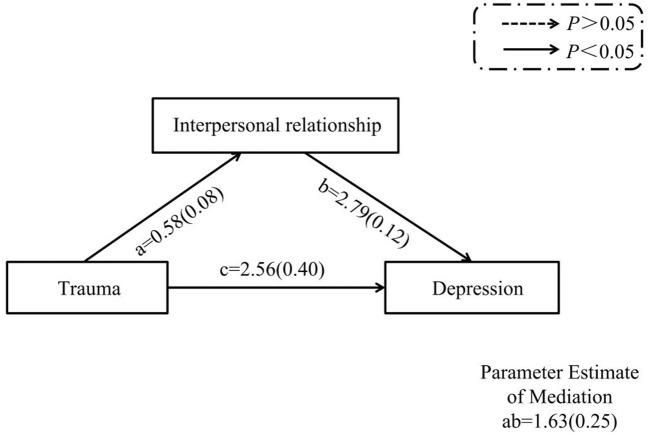
The interaction effects of interpersonal trauma, interpersonal relationship, and depression (mediation analysese).

**Figure 2:**
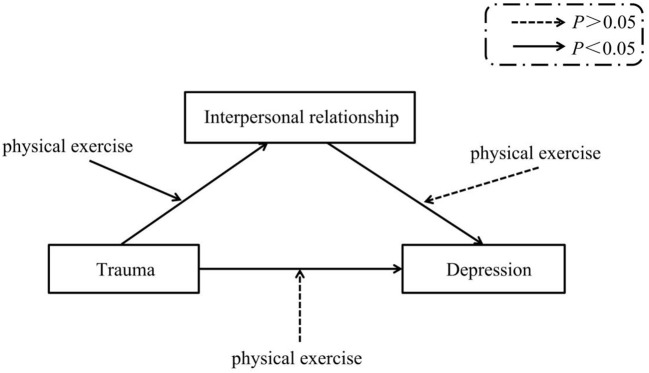
The interaction effects of interpersonal trauma, interpersonal relationship, depression and physical exercise (moderation analyses).

The direct pathway between interpersonal trauma and depression was statistically significant (c = 2.56 (0.40), *p* < 0.05, 95% CI: 1.78–3.34). Separate analyses were conducted to identify the independent mediating effect of interpersonal relationships and the moderating effect of physical exercise in each pathway. The mediation analysis found that the indirect pathway mediated by interpersonal relationships was significant: Interpersonal trauma → interpersonal relationship → depression (ab = 1.63 (0.25), *p* < 0.05, CI: 1.15–2.14). Interpersonal trauma both directly affect risk of depression, and indirectly affect interpersonal relationships through its effect on risk of depression. The moderation analysis found that physical exercise moderated the correlation between interpersonal trauma and interpersonal relationships (*p* < 0.05). No moderating effect from physical exercise on the correlation between interpersonal relationships and depression, or interpersonal trauma and depression was detected (*p* > 0.05). The results of the mediation and moderation analyses are shown in Figures 1, 2, respectively.

## Discussion

This study aimed to investigate the relationship between interpersonal trauma and interpersonal relationships, and physical exercise and the risk of depression in an adolescent population in China using a large school sample. Our findings can be summarized as follows: (1) Interpersonal trauma influences the risk of depression directly; (2) Interpersonal trauma influences the risk of depression indirectly though interpersonal problems; (3) Physical exercise moderates the association between interpersonal trauma and interpersonal relationships.

The results in the present study support there is a significant relationship between interpersonal trauma and depression in adolescents found within previous literature. In a US study, interpersonal trauma was found to be directly associated with depression (37). Previous studies report that interpersonal trauma is furthermore significantly associated with post-traumatic stress disorders (PTSD) (5, 38), which is broadly consistent with the association between depression and trauma in the present study. Future research should consider PTSD as a factor in this population. A study from the US further suggest there are sex differences to interpersonal trauma, reporting that adolescent girls were at an increased risk of depression in their young adulthood when compared to adolescent boys (39). No significant differences in association between interpersonal trauma and depression between male and female adolescents were considered amongst Chinese adolescents. Our findings may differ from those of the previous study as we combined both male and female participants in our sample, future research should consider the sex differences in Chinese adolescents.

The present study found that interpersonal relationships had a mediating effect on the relationship between interpersonal trauma and the risk of depression in Chinese adolescents. These findings support previous studies. More positive relationships with school and family were found to reduce symptoms of depression, and family relationships were identified to affect the association between interpersonal trauma and depression in a US study (37). In a review of the literature, it has been suggested that if parents are physically available and act in a supportive manner this behavior may have a mediating effect of violence on adolescents (40). In contrast, there is an unawareness or misunderstanding by parents on how the exposure of trauma among adolescents might increase the risk of depression. Furthermore, among adolescents who had a more severe experience of violence, they were more likely to feel separation anxiety and have more negative maternal behaviors (41). This supports future research continuing to explore the impact of parental behavior on the risk of depression.

The present study identified physical exercise as a moderator of the relationship between interpersonal trauma and interpersonal relationships in adolescents, with no significant moderating effect on other pathways. Regularly participating in physical exercise was found to weaken the association between interpersonal trauma and interpersonal relationships. In previous studies, physical exercise was found to reduce the risk of depression, however, the direct effect of physical exercise on depression prevention was small (22, 23). With two mechanisms for physical exercise preventing depression in adolescents, the protective effect of physical exercise on depression cannot be ignored. Moreover, physical exercise has been shown to be an effective intervention for adolescent's health (42, 43). Additionally, a study from the US found that adolescents with a higher level of exercise had a better interpersonal relationships and academics (44). The type and length of physical exercise needs to be further categorized in order to explore if certain types and lengths of exercise have more mediating effects toward the risk of depression in Chinese adolescents.

Interpersonal relationships and physical exercise were found to mediate and moderate the association between interpersonal trauma and depression in adolescents, respectively. In this case, besides reducing the risk of depression directly, potential psychological interventions can be designed focusing on interpersonal relationship improvements and school-based physical exercise promotion. Limited studies conducted on depression prevention in Chinese adolescents reveal a weak awareness on depression problems in children and adolescents in China. It therefore is of great importance to promote mental health in children and adolescents in China in order to reduce the risk of mental health disorders. In the UK, mindfulness has been used effectively in schools to improve the mental health of children (30). Furthermore, it has been found that childhood trauma was associated with interpersonal relationships in adults who were had depression (45). However, the long-term mediating effect of interpersonal relationships on the association between trauma and depression was unknown which should be investigated in the future using longitudinal studies.

There are several limitations of the present study. Firstly, although a validated depression scale was used in the study, risk could not be avoided by using a self-reported data collection method. Physical exercise was measured by asking regular participation in physical activity, and not further categorized. Secondly, several questions in the questionnaire (interpersonal trauma) were designed via expert consultation, further validation should be conducted in the future. Thirdly, recall bias is inevitable in a cross-sectional study design, and the data were mainly collected based on self-report of information from the participants. Fourthly, the associations identified in the present study need to be further validated by a large-scale prospective study. Finally, the questionnaire asked about interpersonal trauma within the past 6 months. The implication of this is that it did not consider the impact of incidences of interpersonal trauma that took place before this 6-month period.

This research is exploratory and has led to several potential future lines of research that could be undertaken to better understand the risk of depression in Chinese adolescents. As indicated above, future research could consider the impact of interpersonal trauma which occurred over a longer period of time. A second avenue is that it would be interesting to conduct this study as a longitudinal study, potentially at two 6-month intervals to examine whether there are long term effects. In this case it would also be useful to look at anxiety and PTSD as well as depression. Sex differences should be considered, and type and length of exercise are further areas to research. A final avenue of research could examine the recovery from depression and the impact of interpersonal relationships and physical exercise in this case. Whilst it is important to examine factors associated with depression there is less research done on the recovery from depression and therefore this would be an important avenue for research.

## Conclusions

This is the first study to examine the factors associated with depression in Chinese adolescents with the interaction effects of interpersonal trauma, interpersonal relationships, and physical exercise. A mediating role of interpersonal relationships in the relationship between interpersonal trauma and depression, and a moderating role of physical exercise between interpersonal relationships and depression were identified. Greater interpersonal relationships and regular physical exercise were found to reduce the risk of depression via mediating and moderating roles, respectively. Future prospective studies should be conducted with a more comprehensive physical exercise assessment tool to confirm the findings in the present study as well as potentially look at them cross-culturally. It is crucial to also conduct research developing health promotion interventions focusing on interpersonal relationships and school-based physical exercise in order to reduce the likelihood of depression in the future. Whilst we appreciate there are limitations to our research, the research reported here examines the associations between multiple factors and risk of depression utilizing complex statistical analysis with a large participant sample. We believe our study begins to explore the way in which multiple factors can have an impact on the risk of depression in adolescents.

## Data Availability Statement

The datasets generated for this study are available on request to the corresponding author.

## Ethics Statement

The studies involving human participants were reviewed and approved by The Ethics Committees of Shenzhen Mental Health Center. Written informed consent to participate in this study was provided by the participants' legal guardian/next of kin.

## Author Contributions

JLi, SW, RC, XC, and JLu contributed to the conception and design of the study. JLi, SW, and XC organized the database. RC and KP performed the statistical analysis and wrote the first draft of the manuscript. RC, KP, JLi, YW, AW, MW, and JLu wrote the manuscript. All authors contributed to manuscript revision, read, and approved the submitted version.

### Conflict of Interest

The authors declare that the research was conducted in the absence of any commercial or financial relationships that could be construed as a potential conflict of interest.
